# Neurotoxic Agent-Induced Injury in Neurodegenerative Disease Model: Focus on Involvement of Glutamate Receptors

**DOI:** 10.3389/fnmol.2018.00307

**Published:** 2018-08-29

**Authors:** Md. Jakaria, Shin-Young Park, Md. Ezazul Haque, Govindarajan Karthivashan, In-Su Kim, Palanivel Ganesan, Dong-Kug Choi

**Affiliations:** ^1^Department of Applied Life Sciences, Graduate School, Konkuk University, Chungju, South Korea; ^2^Department of Integrated Bioscience and Biotechnology, College of Biomedical and Health Sciences, Research Institute of Inflammatory Diseases (RID), Konkuk University, Chungju, South Korea; ^3^Nanotechnology Research Center, Konkuk University, Chungju, South Korea

**Keywords:** glutamate receptors, neurodegenerative diseases, neurotoxic agents, nerve tissue, neuronal toxicity

## Abstract

Glutamate receptors play a crucial role in the central nervous system and are implicated in different brain disorders. They play a significant role in the pathogenesis of neurodegenerative diseases (NDDs) such as Alzheimer’s disease, Parkinson’s disease, and amyotrophic lateral sclerosis. Although many studies on NDDs have been conducted, their exact pathophysiological characteristics are still not fully understood. In *in vivo* and *in vitro* models of neurotoxic-induced NDDs, neurotoxic agents are used to induce several neuronal injuries for the purpose of correlating them with the pathological characteristics of NDDs. Moreover, therapeutic drugs might be discovered based on the studies employing these models. In NDD models, different neurotoxic agents, namely, kainic acid, domoic acid, glutamate, β-*N*-Methylamino-L-alanine, amyloid beta, 1-methyl-4-phenyl-1,2,3,6-tetrahydropyridine, 1-methyl-4-phenylpyridinium, rotenone, 3-Nitropropionic acid and methamphetamine can potently impair both ionotropic and metabotropic glutamate receptors, leading to the progression of toxicity. Many other neurotoxic agents mainly affect the functions of ionotropic glutamate receptors. We discuss particular neurotoxic agents that can act upon glutamate receptors so as to effectively mimic NDDs. The correlation of neurotoxic agent-induced disease characteristics with glutamate receptors would aid the discovery and development of therapeutic drugs for NDDs.

## Background

Glutamate receptors are the most abundant type of excitatory neurotransmitter receptors, and they are involved in mediating common excitatory synaptic transmissions in the brain and spinal cord (Jensen, 2009; Byrne et al., 2014). These receptors are very complex in nature and more than 20 different glutamate receptors have been recognized in the mammalian central nervous system (Altevogt et al., 2011). Glutamate receptors are categorized into two groups: ionotropic and metabotropic. iGluRs are voltage-sensitive, whereas mGluRs are ligand-sensitive (Altevogt et al., 2011). The subtypes of iGluRs are named for the chemical agonists that selectively bind to these subfamily members: NMDARs, AMPARs, and KARs (Conn and Pin, 1997). These subtypes have different subunits: the NMDAR subunits are GluN1, GluN2A, GluN2B, GluN2C, GluN2D, GluN3A, and GluN3B; the AMPAR subunits are GluA1, GluA2, GluA3, and GluA4; and the KAR subunits are GluK1, GluK2, GluK3, GluK4, and GluK5 (Tang et al., 2004). According to their pharmacological and signal transduction properties, mGluRs are divided into three broad groups that comprise eight subtypes (Jia et al., 2014). Group I and II mGluRs have two subunits: mGluR1 and mGluR5, and mGluR2 and mGluR3, respectively. Group III mGluRs have four subunits: mGluR4, mGluR6, mGluR7, and mGluR8 (Wong et al., 2005).

Glutamate receptors are best known for their mediation of glutamate in learning and memory through plasticity, that is the modification of channel properties; enhanced glutamate neurotransmission; and gene expression (Barco et al., 2006). They are involved in the pathogenesis of a variety of neurological disorders, including anxiety, schizophrenia, and epilepsy. (Rubio et al., 2012; Barker-Haliski and White, 2015; De Filippis et al., 2015). In addition to these disorders, various studies have reported that iGluRs and mGluRs play crucial roles in the pathogenesis of NDDs, such as AD, PD, Huntington’s disease (HD), amyotrophic lateral sclerosis (ALS), multiple sclerosis, and spinocerebellar ataxia (Geurts et al., 2003; Jin and Smith, 2007; O’Neill and Witkin, 2007; Fernandes and Raymond, 2009; André et al., 2010; Fallarino et al., 2010; Kwak et al., 2010; Ahmed et al., 2011; Spalloni et al., 2013; Titulaer et al., 2014; van Beugen et al., 2014; Iizuka et al., 2015; Guntupalli et al., 2016; Ishibashi et al., 2016; Zhang Y. et al., 2016; Ribeiro et al., 2017).

Based on the complexity of the mechanistic progression of NDDs, elucidating the proper disease pathophysiology and therapeutics of NDDs remains a major challenge. Recently, many neurotoxic agents have been employed in experiments in order to explore cellular functions and dysfunctions (Kumar and Kumar, 2010; van der Star et al., 2012; Abbasi et al., 2013; Jiang et al., 2015; Parekh, 2015; Ahmad et al., 2017; Kim et al., 2017). Their correlation with pathological characteristics of diseases via the utilization of neurotoxic agent-induced models is a helpful way to screen and discover potential therapeutic drugs for NDDs. Prior studies have also documented the actions of common neurotoxic agents that cause injury in NDD models (Schober, 2004; Bové et al., 2005; Hisahara and Shimohama, 2011; More et al., 2016). With respect to diverse neuronal functions, we focus on glutamate receptors as a target in neurotoxic agent-induced injury that may aid in the discovery of therapeutic drugs specifically for NDDs.

## Glutamate Receptors as Potential Targets in Neurotoxic Agent-Induced NDD Models

### Agents That Act on iGluRs and mGluRs

#### Glutamate, Kainic Acid, and Domoic Acid

Glutamate, a major excitatory neurotransmitter in the central nervous system, plays a vital role in neuronal cell differentiation, migration, and survival in the developing brain, basically via facilitating the entry of Ca^2+^ (Llorente-Folch et al., 2016). Kainic acid (KA), an agonist for the iGluR subtype which is known as a non-degradable analog of the excitotoxin, glutamate, offers a well-characterized model for the study of NDDs (Wang et al., 2005; Zhang et al., 2010; Zheng et al., 2010). DomA, a naturally occurring marine neurotoxin produced by members of the diatom genus, *Pseudo-nitzschia*, is a structural relative of KA (Lu et al., 2013).

Glutamate is capable of binding to and activating both iGluRs and mGluRs. In the brain, intracellular glutamate concentrations are in the millimolar range, and extracellular glutamate concentrations remain in the low micromolar range. These concentrations are achieved through the action of excitatory amino acid transporters that import glutamate and aspartate into astrocytes and neurons. Excess extracellular glutamate may lead to excitotoxicity *in vitro* and *in vivo* through the overactivation of iGluRs (Lewerenz and Maher, 2015). In general, glutamate triggers neuroinflammation while glutamate-induced excitotoxicity may contribute to neuronal cell death in NDDs (Lee et al., 2017). Glutamate-induced excitotoxicity causes cell death, apoptosis, and autophagy in both hippocampal cells (HT22) and primary cultured hippocampal neuron cells with neurotoxicity in differentiated Y-79 cells, BV-2 cells, and PC12 cells. Mitochondrial dysfunction, oxidative damage, and neuroinflammation are also key toxic effects in the glutamate-induced neurotoxicity model (Bak et al., 2016; Shinoda et al., 2016; Wang K. et al., 2016; Xu et al., 2016; Chen Z.W. et al., 2017). Numerous studies have described glutamate-induced neurotoxicity through the action of glutamate receptors. In human embryonic stem cell-derived neurons, glutamate produces NMDAR-dependent excitotoxicity. On the other hand, an NMDAR antagonist reduces glutamate-induced Ca^2+^ influx, which leads to the reduction of excitotoxicity (Gupta et al., 2013). In addition, berberine-induced mitochondria and NMDAR-dependent toxicity sensitize neurons to glutamate injury. Memantine (an NMDARs antagonist) and dizocilpine (MK-801) (a non-competitive NMDARs antagonist) completely block berberine-induced neurotoxicity (Kysenius et al., 2014). Another study found that MK-801 and γ-D-glutamylaminomethyl sulfonic acid (a KARs/AMPARs antagonist) wholly prevents glutamate-induced impairment in hippocampal cells. An p38 MAPK inhibitor, SB203580, also prevents glutamate-induced cell damage, but an MEK1 inhibitor, PD98059, does not alter glutamate-induced cell death in the intracellular signaling pathways (Molz et al., 2008). According to the most recent research on glutamate-induced toxicity in differentiated PC12 cells, the glutamate-induced dysfunction of Ca^2+^ homeostasis is protected by FAM3A upregulation. This activity is accomplished through the inhibition of mGluR1/5-dependent Ca^2+^ release by the endoplasmic reticulum (ER) and attenuation of the stromal interaction molecule-1 (STIM1)-Orai1 channel interactions that modulate store-operated Ca^2+^ entry (Song et al., 2017). Further, mGluR5 is expressed on astrocytes and through its activation, aquaporin 4-mediated glutamate-induced neurotoxicity causes partial mediation of astrocyte swelling. An mGluR5 agonist, (S)-3,5-dihydroxyphenylglycine (DHPG), which activates mGluR5 in cultured astrocytes, mimics the effect of glutamate. Incubation of DHPG with fenobam (an mGluR5 antagonist) negates this, and DL-threo-β-benzyloxyaspartic acid (DL-TBOA), a glutamate transporter inhibitor, does not abolish this agonistic effect (Shi et al., 2017).

The KA-induced neurotoxicity model is suitable for both *in vivo* and *in vitro* studies using rodents and several cell lines, such as BV-2 microglia, PC12 cells, and SH-SY5Y cells (Zhang et al., 2010; Hsieh et al., 2011; Xie et al., 2011; Luo et al., 2013; Nampoothiri et al., 2014; Nabeka et al., 2015). By acting on KARs, KA causes neuroexcitotoxic and epileptogenic properties. KA induces behavioral changes in rodents and causes a variety of cellular events to take place, including the influx of cellular Ca^2+^, neuroinflammation, production of reactive oxygen species (ROS) and mitochondrial dysfunction. It eventually leads to neuronal apoptosis and necrosis in many regions of the brain, particularly in the hippocampal subregions, cornu ammonis 1 (CA1), cornu ammonis 3 (CA3), and hilus of dentate gyrus (Wang et al., 2005; Zhang et al., 2010; Xie et al., 2011; Nabeka et al., 2015). Moreover, in cellular models, KA produces effects similar to those seen in rodent models (Hsieh et al., 2011; Nampoothiri et al., 2014). According to a recent study involving KA-induced excitotoxic hippocampal neuronal damage in rats, 2-Methyl-6-(phenylethynyl)-pyridine (a negative allosteric modulator of mGluR5) and LY354740 (an agonist of mGluR2) treatments ameliorate KA-induced neuronal cell death. Based on these results, both KARs and mGluRs may be involved in the KA-induced neuronal toxicity (Pershina et al., 2017).

**FIGURE 1:**
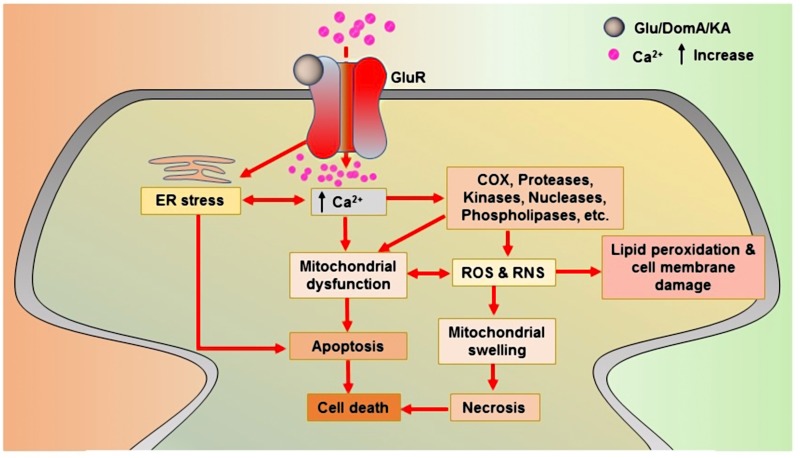
Glutamate receptor-mediated neurotoxic actions of glutamate, domoic acid, and kainic acid. Upon binding to glutamate receptors, all toxins produce an agonistic reaction that lead to cell death. Glu: Glutamate; KA: Kainic acid; DomA: Domoic acid; COX: Cyclooxygenase; ROS: Reactive oxygen species; and RNS: Reactive nitrogen species.

As a KAR agonist, DomA is considered a potent neurotoxin and is used in experimental models to cause neurotoxicity. DomA-induced neurotoxicity causes neuroinflammation, mitochondrial dysfunction, oxidative stress, apoptosis, cognitive impairment, and neuronal cell death (Ananth et al., 2003; Chandrasekaran et al., 2004; Giordano et al., 2009; Lu et al., 2013; Wang D. et al., 2016). It is also employed in order to induce the symptoms of epilepsy in animal models (Buckmaster et al., 2014). The modulation of iGluRs may play a part in DomA-induced excitotoxicity (Qiu et al., 2005). In a neonatal rat model, a very low dose of DomA was shown to elicit a conditioned odor preference, and this was partly attributed to NMDARs involvement (Tasker et al., 2005). According to another investigation, AMPARs/KARs primarily regulate the neurotoxic effects of DomA. NBQX (a AMPARs/KARs antagonist) completely prevents DomA-induced toxic effects, whereas the NMDARs antagonist, (2*R*)-amino-5-phosphonopentanoate (APV), only partially blocks these effects (Hogberg and Bal-Price, 2011). The glutamate-, DomA-, and KA-induced progressions of major neurotoxicity via glutamate receptors are depicted in **Figure 1**.

#### 1-Methyl-4-phenyl-1,2,3,6-tetrahydropyridine and 1-Methyl-4-phenylpyridinium

1-methyl-4-phenyl-1,2,3,6-tetrahydropyridine (MPTP) is a common neurotoxic agent used to induce PD in animal models. MPTP yields large variations in nigral cell loss, striatal dopamine (DA) loss, and behavioral deficits (Meredith and Rademacher, 2011). 1-methyl-4-phenylpyridinium (MPP^+^), a metabolite of MPTP by monoamine oxidase-B, is also considered to be a neurotoxic agent and is also commonly used in both *in vitro* and *in vivo* PD models (Gasparini et al., 2013). MPTP activates the NMDARs and increases glutamate release in the striatum, which causes a large influx of Ca^2+^-induced neuronal excitotoxicity (Wang et al., 2010). The modulation of glutamate receptors by MPTP may be responsible for producing the associated neurotoxic effects. In addition, certain iGluRs antagonists have shown antiparkinsonian and anti-dyskinetic activities (Gasparini et al., 2013). Moreover, MPTP upregulates mGluR5 in monkey model. Chronic treatment with an mGluR5 antagonist, (3-[(2-methyl-1,3-thiazol-4-yl) ethynyl] pyridine) (MTEP), significantly protects dopaminergic and noradrenergic neurons from MPTP-induced toxicity (Samadi et al., 2008; Masilamoni et al., 2011). MPP^+^ interacts with NMDARs as a partial agonist and may dysregulate receptor functioning. Concerning the available research, NMDAR antagonists inhibit the actions of MPP^+^, specifically by decreasing MPP^+^-induced mitochondrial dysfunction (Camins et al., 1997). In a study, an mGluR8 agonist, (S)-3,4-Dicarboxyphenylglycine, demonstrates neuroprotective activity against MPP^+^-induced cell death in SH-SY5Y cells (Jantas et al., 2014).

#### Rotenone

Rotenone is a commonly used pesticide and fish toxin that impedes mitochondrial respiratory chain complex I (Zhang et al., 2006). It a valuable tool for PD research and exposure to rotenone causes the induction of parkinsonism in rodents. Nowadays, rotenone is an extensively used toxin to induce neurotoxicity in both *in vitro* and *in vivo* models of PD. It causes aging-related SN dopaminergic neurodegeneration in rats (Wang X. et al., 2015). Rotenone potently augmented NMDA-evoked currents in rat DA neurons through a tyrosine kinase-dependent mechanism. Further study showed that the potentiation of NMDA currents by a tyrosine kinase-dependent process attenuates the voltage-dependent Mg^2+^ block of NMDA-gated channels (Wu and Johnson, 2009). In addition, rotenone modulates mGluRs as it damages DNA through mGluR5. The selective mGluR5 antagonist protects rotenone-induced neurotoxicity by mitigating the oxidative stress-related damage to DNA associated with 8-hydroxy-2′-deoxyguanosine production, and also decreases the phosphorylation of extracellular-signal-regulated kinase activity and thioredoxin-2 expression (Xia et al., 2015).

#### Methamphetamine

As a sympathomimetic amine, METH belongs to the phenethylamine and amphetamine classes of psychoactive drugs. It is abused extensively for its euphoric, stimulant, empathogenic, and hallucinogenic properties (Yu et al., 2015). As a neurotoxic agent, METH can be used to induce neurotoxicity in a study model, which may be helpful in the study of NDDs, particularly in study of PD. METH-induced neurotoxicity is characterized by a long-lasting depletion of striatal DA and serotonin as well as by damage to striatal dopaminergic and serotonergic nerve terminals (Kita et al., 2003). The principal neurotoxic effects caused by METH are oxidative stress, neuroinflammation, and apoptosis leading to neuronal cell death (Choi et al., 2002; Raineri et al., 2012; Shin et al., 2012; Jayanthi et al., 2014; Jumnongprakhon et al., 2014). Studies have reported that METH downregulates the glutamate receptors. In the hippocampus, METH induction decreases the permeability and/or functionality of NMDARs and AMPARs, impairing spatial working memory (Simoes et al., 2007). In the rat striatum and frontal cortex, METH changes the NMDAR and AMPAR subunit levels (Simões et al., 2008). Besides, the cortical iGluR antagonism protects against METH-induced striatal neurotoxicity (Gross et al., 2011). A few studies have correlated METH-induced neurotoxicity with mGluRs. Antagonism of mGluR1 by its selective antagonist JNJ16259685 [(3,4-dihydro-2H-pyrano-[2,3-b]quinolin-7-yl)-(cis-4-methoxycyclohexyl) metha-none] attenuates cocaine- and METH- treated behavioral effects in squirrel monkeys (Achat-Mendes et al., 2012). In addition to mGluR1, mGluR5 receptors play an important role in METH reinforcement and METH-seeking behavior. An mGluR5 antagonist, 2-methyl-6-(phenylethynyl)-pyridine, dose-dependently reduces the reinforcing effects of METH under a fixed-ratio 1 and a progressive ratio schedule of reinforcement without altering overall responding for food. It also dose-dependently prevents the cue and drug-induced reinstatement of METH-seeking behavior (Gass et al., 2009). Another study reported that mGluR5 plays a role in the maintenance of place preference memory and that its negative allosteric modulators could be potentially used in METH addiction therapy (Herrold et al., 2013).

#### Amyloid Beta

**FIGURE 2:**
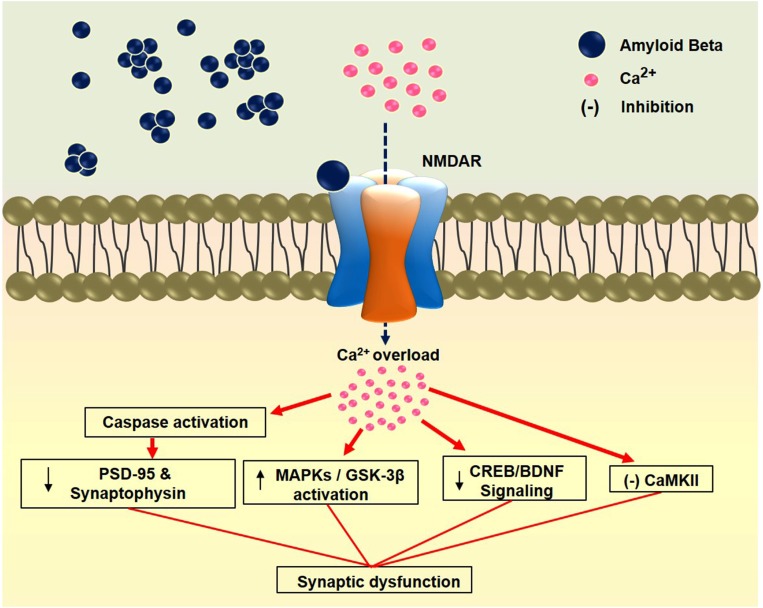
Amyloid beta-induced synaptic dysfunction through NMDAR. Binding of Aβ to the NMDAR causes calcium overload in the synapse leading to impairment in various signaling pathways. Finally, these impairments cause synaptic dysfunction. PSD-95: Postsynaptic density protein 95; MAPKs: Mitogen-activated protein kinases; GSK-3β: Glycogen synthase kinase-3 beta; CREB: cAMP-response element binding protein; BDNF: Brain-derived neurotrophic factor; CaMKII: Calcium/calmodulin-dependent protein kinase II

Amyloid beta (Aβ) acts as a neurotoxic agent by initiating biochemical cascades that ultimately lead to synaptotoxicity and neurodegeneration (Walsh and Selkoe, 2004). It can interrupt excitatory synaptic transmission and plasticity in the brain via the dysregulation of AMPARs and NMDARs (Guntupalli et al., 2016). Recent research employing Aβ-induced neurotoxicity models have shown that the altered activity of NMDARs plays a major role in disease pathogenesis. Aβ enhances the activation of extrasynaptic NMDARs by decreasing neuronal glutamate uptake and inducing glutamate spillover (Wang Z.-C. et al., 2013). It binds to hippocampal neuron NMDAR subunits GluN1 and GluN2B (Lacor et al., 2004; Lacor et al., 2007). The GluN2B subunit is involved in regulating the action of Aβ oligomers by increasing intracellular Ca^2+^ in dendritic spines, resulting in the reduction of dendritic spine and synaptic density, which leads to early synaptic dysfunction (Shankar et al., 2007). In an Aβ-induced model, synaptic alterations were mitigated by blocking glutamate from binding to NMDARs though the use of an NMDARs antagonist (Birnbaum et al., 2015). Correspondingly, the stimulation of GluN2B by Aβ oligomer triggers the activation of MAPK and the subsequent downregulation of cAMP response element-binding protein (CREB) (Li et al., 2011). Indeed, Aβ reduces BDNF signaling by impairing axonal transport, which leads to synaptic dysfunction (Poon et al., 2011). A recent report showed that inhibition of NMDARs prevents the Aβ-induced loss of BDNF function (Tanqueiro et al., 2018). In addition, through in an α7-nicotinic acetylcholine receptor (α7-nAChR)-dependent manner, Aβ oligomers induce the endocytosis of NMDARs (Snyder et al., 2005). Likewise, α7-nAChRs are linked to the deregulation of NMDA signaling pathways (Roselli et al., 2005; Shankar et al., 2007). Interestingly, relatively low doses of NMDA-antagonists have been shown to reverse Aβ-induced synaptic disruption (Li et al., 2011; Rönicke et al., 2011). Aβ downregulates the caspase-mediated loss of two synaptic proteins, PSD-95 and synaptophysin. It suppresses NR2A function and activates NR2B following the induction of caspase-8 and caspase-3 activities. On the other hand, MK-801 and ifenprodil (an NR2B antagonist) prevent Aβ-induced toxicity (Liu et al., 2010). In hippocampal neurons, Aβ oligomers disrupt axonal transport initiated by NMDAR-dependent mechanisms, and this is modulated by the enzyme glycogen synthase kinase-3β (Decker et al., 2010). In another study, the activation of Aβ-induced striatal-enriched protein tyrosine phosphatase was shown to lead to the dephosphorylation of tyrosine residues on NMDARs. Dephosphorylation of the GluN2B subunit correlates with increased NMDARs endocytosis and suppression of its synaptic function. On the other hand, reelin activates Src family tyrosine kinases and enhances tyrosine phosphorylation of the GluN2A and GluN2B subunits. Reelin signaling may prevent Aβ-induced NMDARs endocytosis and Src family tyrosine kinases activation (Durakoglugil et al., 2009). A proposed mechanism of Aβ-induced impairment in major types of signaling through NMDARs that leads to synaptic dysfunction is presented in **Figure 2**.

Aβ-induced synaptic dysfunction has been attributed to the synaptic removal of AMPARs. Aβ-induced change in the subcellular distribution of Ca^2+^/calmodulin-dependent protein kinase II may drive the removal of AMPARs from the synaptic membrane by Aβ (Gu et al., 2009). Aβ initiates synaptic and memory deficits by removing GluA3-containing AMPARs from synapses (Reinders et al., 2016). It disrupts mitochondrial trafficking, which may contribute to AMPAR removal as well as trafficking defects that cause synaptic inhibition (Rui et al., 2010). Furthermore, Aβ-induced dendritic spine loss and reductions in pre- and post-synaptic protein levels in hippocampal slice cultures can impair hippocampal LTP (Zhang et al., 2009; Wang Z.-C. et al., 2013; Birnbaum et al., 2015).

Aβ-induced ectopic NMDA and mGluR-mediated signaling coupled with caspase-3 activation may inhibit LTP and also facilitate long-term depression (LTD) (Hu et al., 2012). Within the synaptic space, membrane-bound Aβ oligomers accumulate and via a lateral diffusion process, gradually aggregate in order to form large non-mobile clusters. Aβ pathological clusters form complexes with mGluR5 receptors, which decreases the mobility of mGluR5 and causes its anomalous accumulation at the postsynaptic membrane. This is followed by calcium deregulation, synaptic disruption, and loss of NMDARs (Renner et al., 2010). In a transgenic model study, Aβ oligomer-cellular prion protein complexes activated mGluR5 at the neuronal surface, which led to the disruption of neuronal function (Um et al., 2013). The downregulation of mGluR and desensitization of the frontal cortex in AD patients correlated with AD-related neuropathological variations. Furthermore, the chronic activation of mGluR5 increased NMDA-dependent Aβ neurotoxicity, whereas mGluR5 antagonism exhibited neuroprotective effects against Aβ excitotoxic processes and prevented impairments in learning, memory and synaptic density (Rammes et al., 2011; Um et al., 2013).

#### Homocysteine

Homocysteine (Hcy), a sulfur-containing amino acid derived from the metabolism of methionine, is an independent risk factor for AD (Ataie et al., 2010c; Li et al., 2016). As a known neurotoxic agent, Hcy is used to induce neurotoxicity. In several animal model studies, Hcy brought about synaptic dysfunction, oxidative stress, neurochemical imbalance, and apoptosis, resulting in cognitive impairment and neuronal cell death. Thus, an Hcy-induced neurotoxicity model might be suitable for the study of AD (Ataie et al., 2010a,b,c; Wei et al., 2014; Kamat et al., 2016; Li T. et al., 2017). Hcy has been shown to modulate glutamate receptors, which leads to various neurotoxic effects. The activation of Hcy-NMDAR-mediated extracellular signal-regulated kinase causes neuronal cell death. Further, it modulates hippocampal glutamate and the NMDAR/AMPAR ratio in a rat model of chronic unpredictable mild stress-induced depression (Poddar and Paul, 2009; Liu et al., 2013; Moustafa et al., 2014; Poddar et al., 2017). Moreover, Hcy modulates mGluRs. An mGluR1 antagonist produces neuroprotective effect in the Hcy-induced neurodegenerative model (Yeganeh et al., 2013).

#### β-*N*-methylamino-L-alanine

The non-proteinogenic amino acid β-*N*-methylamino-L-alanine (BMAA), was first identified in the seeds of *Cycas micronesica* in 1967 (McCarron et al., 2014), though a wide range of cyanobacteria are now known to produce BMAA. Recently, the most common group of algae (diatoms) was also found to produce it (Delzor et al., 2014). BMAA is considered one of the first environmental factors that contributes to the etiologies of AD, PD, and ALS (Zhou et al., 2010). It is a common neurotoxin utilized in the study of neurodegeneration in cellular and animal models, specifically those for the study of ALS/Parkinsonism-dementia complex. BMAA causes neuroinflammation, oxidative stress, apoptosis and cognitive impairment (Brownson et al., 2002; Lobner, 2009; Santucci et al., 2009; Zhou et al., 2010; Muñoz-Saez et al., 2013; Al-Sammak et al., 2015; Takser et al., 2016; Laugeray et al., 2017; Petrozziello et al., 2017). It elicits neurotoxicity by acting as an agonist for glutamate receptors such as AMPARs/KARs, NMDARs, and mGluR5 (Lobner, 2009; Delzor et al., 2014). BMAA causes a significant increase in Ca^2+^ influx and enhanced ROS production, while also disrupting mitochondrial activity in rat olfactory ensheathing cells (Chiu et al., 2013). In addition, it interferes with neurotransmission in human neuroblastoma cells. BMAA alters alanine, aspartate, and glutamate metabolism and also modifies numerous neurotransmitters/neuromodulators, such as GABA and taurine (Engskog et al., 2017). However, the mechanism of BMAA-induced neurotoxicity is not yet fully understood, and its role as a glutamate receptor agonist may in fact lead to excitotoxicity that hampers glutamate transport systems (Zimmerman et al., 2016).

#### 3-Nitropropionic Acid

3-Nitropropionic acid (3-NP) is a common neurotoxic agent used to study HD and is an irreversible inhibitor of mitochondrial complex-II. In the experimental animal model, it caused mitochondrial dysfunction, oxidative stress, biochemical imbalance, neuroinflammation, apoptosis, and autophagy, leading to neuronal cell death (Binawade and Jagtap, 2013; Solesio et al., 2013; Shetty et al., 2015; Jamwal and Kumar, 2016; Thangarajan et al., 2016). 3-NP was shown to produce neurotoxicity via the modulation of glutamate receptors. One study has suggested that 3-NP produces a neurotoxic effect through GluN2B-containing NMDARs (Centonze et al., 2006). In addition, the glutamate receptor antagonist, (2*R*)-amino-5-phosphonovaleric acid (AP5), negates the 3-NP-induced NMDAR-mediated second peak in ROS, mitochondrial fission, and cell death (Liot et al., 2009). The cannabinoid agonist WIN55,212-2 has been shown to produce a neuroprotective effect against 3-NP-induced striatal neurotoxicity via the induction of NMDARs hypofunction (Maya-López et al., 2017). Moreover, mGluR5 may be involved in 3-NP-induced neurotoxicity. In a study, MTEP produced neuroprotective activity in a 3-NP-induced neuronal injury model (Souza et al., 2014).

#### Cuprizone

Cuprizone is a common neurotoxic agent indicated to induce neurotoxicity to study NDDs. It is particularly used to induce multiple sclerosis-like syndromes. In the experimental animal model, it has shown to cause demyelination, oxidative stress, neuroinflammation, and apoptosis leading to neuronal cell death (Gudi et al., 2014; Zimmermann et al., 2014; Slowik et al., 2015; Sághy et al., 2016; Ragerdi Kashani et al., 2017; Sanadgol et al., 2017). In the cuprizone model of demyelination, the NMDARs specific antagonist MK-801 delays remyelination. NMDARs plays a critical role in the regulation of oligodendrocyte precursor cell differentiation *in vitro* and remyelination in cuprizone model, which may provide a potential target for the treatment of demyelination disease (Li et al., 2013). Another study reported that cuprizone treatment affected glutamate-receptors and -transporters differently in the gray and white matter areas of the brain, specifically, showed that it regulates the glutamate-aspartate transporter (*Slc1a3*) and neutral amino acid transporter A (*Slc1a4*) genes compared with other genes. Among the different NMDAR subunits, GluN2A was upregulated in the demyelinated corpus callosum (CC) and mGluR2 was downregulated in the demyelinated gray matter (Tameh et al., 2013).

#### Doxorubicin

Doxorubicin (DOX) is a known neurotoxic agent suitable for both *in vivo* and *in vitro* studies. In experimental models, DOX induction of neurotoxicity causes mitochondrial dysfunction, oxidative stress, neuroinflammation, apoptosis, cognitive impairments, and neuronal cell death (Shokoohinia et al., 2014; Siswanto et al., 2016; Cheruku et al., 2017). Several molecular studies have shown that DOX impairs glutamate receptors, leading to neuronal toxicity. In a DOX-induced neurotoxicity study, memantine was shown to counter neuronal cell death by blocking NMDARs (Jantas and Lason, 2009). In addition, mGluR II and III activators produced neuroprotective effects on DOX-induced cellular injury (Jantas et al., 2015). Furthermore, LTP and AMPARs impairment has been shown in DOX-treated animals. Compared to controls, the expression of the AMPAR subunit, GluA1, was significantly decreased, whereas the expression of the GluA2 subunit was significantly elevated (Alhowail, 2014). Another study showed that NMDARs and AMPAR might be involved in DOX-induced damage to DNA in neurons (Manchon et al., 2016).

#### Glucocorticoid

The hormone, GC, rises in concentration response to stress, which can cause neuronal loss (Kim et al., 2010). GC-induced neurotoxicity might be an appropriate model for the study of NDDs. It causes oxidative stress, memory impairment, neuronal cell death, and apoptosis (De Quervain et al., 2003; Lu et al., 2003; Roozendaal et al., 2003; You et al., 2009; Xu Y. et al., 2013) the latter through iGluRs and mGluRs (Lu et al., 2003). The stress-induced elevation of GC increases microglia proliferation through the activation of NMDARs. MK-801 has been shown to prevent the increase of microglia following the administration of exogenous GC corticosterone (Nair and Bonneau, 2006).

#### Harmaline

Harmaline-induced neurotoxicity in animals also could be a robust model for the study of NDDs (Iseri et al., 2011; Dahmardeh et al., 2017). Harmaline modulates both iGluRs and mGluRs (Kolasiewicz et al., 2009; Iseri et al., 2011). As an inverse agonist of NMDARs, it produces tremors (Du et al., 1997). The synchronous activation of the olivocerebellar pathway and release of glutamate in the cerebellum (which acts on NMDARs and AMPARs) is proposed to be main cause of harmaline-induced tremors (Shourmasti et al., 2014). In addition, the agonistic action of harmaline on mGluR1 produces motor disturbances (Kolasiewicz et al., 2009). On the other hand, memantine produces neuroprotective and anti-tremorgenic activities against harmaline-induced tremors and neurodegeneration (Iseri et al., 2011).

#### Pentylenetetrazol

The tetrazole derivative, PTZ, causes convulsions in mice, rats, cats, and primates, likely by interfering with GABA-mediated inhibition. PTZ is best known for its use in the screening of antiepileptic drugs (Zhao, 2006). It causes neuroinflammation and oxidative stress, which affects cognition. In addition, PTZ has been observed to cause amnesia in animal models (Oscós-Alvarado and de Muñoz, 1981; Pourmotabbed et al., 2011; dos Santos Branco et al., 2013). It is also known to modulate glutamate receptors. In a PTZ-induced kindling model of epilepsy, NMDARs are upregulated in both the hippocampus and cortex. KARs/AMPARs antagonists act as anticonvulsants against the tonic hind limb component of PTZ-induced seizures in developing rats (Velíšek et al., 1995; Ekonomou and Angelatou, 1999; Rogawski, 2011). The group III mGluR agonist CPPG [(RS)-*α*-cyclopropyl-4-phosphonophenylglycine] has been shown to attenuate PTZ-induced seizures. Moreover, it increases glutamate concentrations in the hippocampus of non-kindled control rats (Maciejak et al., 2009). The effects of several neurotoxic agents on both iGluRs and mGluRs are listed in **Table 1**.

**Table 1:** Major impairing effects of several neurotoxic agents on iGluRs and mGluRs.

Toxic agents	Major modulating effects	Reference
MPTP	Upregulates mGluR5 and causes damages in dopaminergic and noradrenergic neurons	Samadi et al., 2008; Masilamoni et al., 2011
MPP^+^	Activates NMDARs and dysregulates mGluR8; causes mitochondrial damage and neuronal cell death	Camins et al., 1997; Jantas et al., 2014
Rotenone	Potentiates NMDA current; activates mGluR5; causes excitotoxicity and DNA damage	Wu and Johnson, 2009; Xia et al., 2015
METH	Impairs NMDARs, AMPARs, mGluR1, and mGluR5; causes striatal neurotoxicity and behavioral dysfunction	Simões et al., 2008; Gass et al., 2009; Gross et al., 2011; Achat-Mendes et al., 2012
Homocysteine	Activates NMDARs and mGluR1; causes neuronal cell death	Poddar and Paul, 2009; Yeganeh et al., 2013
BMAA	Activates AMPARs/KARs, NMDA and mGluR5 receptors; causes excitotoxic damage	Lobner, 2009; Delzor et al., 2014
3-NP	Activates NMDARs and mGluR5; causes ROS elevation, mitochondrial fission, and cell death	Liot et al., 2009; Souza et al., 2014
Cuprizone	Activates NMDARs; causes demyelination; affects glutamate-receptors and -transporters differently; downregulates mGluR2	Li et al., 2013; Tameh et al., 2013
Doxorubicin	Dysregulates NMDARs, AMPARs, and mGluR II and III; causes neuronal injury	Jantas and Lason, 2009; Jantas et al., 2015; Manchon et al., 2016
Glucocorticoid	Activates NMDARs and mGluR III; causes microglia proliferation and apoptosis	Lu et al., 2003; Nair and Bonneau, 2006
Harmaline	Activates NMDARs, AMPARs, and mGluR1; produces tremors	Kolasiewicz et al., 2009; Shourmasti et al., 2014
PTZ	Dysregulates NMDARs, AMPARs, and mGluR III; causes seizures	Velíšek et al., 1995; Ekonomou and Angelatou, 1999; Maciejak et al., 2009; Rogawski, 2011

*3-NP: 3-Nitropropionic Acid; BMAA: β-N-methylamino-L-alanine; MPTP: 1-methyl-4-phenyl-1,2,3,6-tetrahydropyridine; MPP^+^: 1-methyl-4-phenylpyridinium; METH: Methamphetamine; and PTZ: Pentylenetetrazol.*

### Agents That Act on iGluRs

#### Ethanol

Chronic exposure to ethanol has complex and long-lasting effects on the function and expression of innumerable neuroreceptors as well as their modulators (Lovinger, 1997). Ethanol-induced neurotoxicity models have previously been used to study NDDs (Naseer et al., 2014; Saito et al., 2016). *In vivo* and *in vitro* studies have shown that chronic ethanol exposure elevates *GluN1* and *GluN2B* gene expression and their polypeptide levels. In animal models, ethanol-induced neurotoxicity has been shown to cause oxidative stress, apoptosis, and neuroinflammation, ultimately causing neurodegeneration (Ullah et al., 2012, 2013; Naseer et al., 2014; Tajuddin et al., 2014; Ahmad et al., 2016; Wang P. et al., 2017). Ethanol has been shown to produce neurotoxicity in cellular studies on BV-2 microglia, PC12 cells, and HT22 cells (Casañas-Sánchez et al., 2016; Zhang J. et al., 2016; Huang et al., 2017). As a potent inhibitor of NMDARs, it acts on glutamate receptors and impairs the functionality of NMDARs and AMPARs. Moreover, ethanol inhibits glutamate receptor-mediated synaptic plasticity such as NMDA-dependent LTP (Wirkner et al., 1999; Möykkynen et al., 2003, 2009; Hicklin et al., 2011; Möykkynen and Korpi, 2012; He et al., 2013). The effect of alcohol and molecular changes within the regulatory process that modulates NMDARs functions, including factors that alter transcription, translation, post-translational modifications, and protein expression along with those that influence their interactions with different regulatory proteins (downstream effectors) constantly increases at the cellular level. Epigenetic dimension (i.e., histone modification-induced chromatin remodeling and DNA methylation that occurs in the process of alcohol-related neuroadaptation) is a key molecular mechanism in alcohol-mediated NMDARs alteration (Chandrasekar, 2013).

#### Ammonia

An ammonia-induced neurotoxicity model uncovered potential therapeutic strategies (Hadjihambi et al., 2014). Elevated ammonia levels damage motor neurons through numerous means, including endoplasmic reticulum (ER) stress, cyclin-dependent kinase 5 activation, macroautophagy-endolysosomal pathway impairment, oxidative and nitrosative stress, neuronal hyperexcitability, and neuroinflammation (Parekh, 2015). In order to produce neurotoxicity, ammonia modulates glutamate receptors and activates NMDARs which in turn leads to the following: (i) adenosine triphosphate (ATP) is depleted in the brain, causing glutamate release; (ii) calcineurin and dephosphorylation are activated and Na^+^/K^+^-ATPase is activated in the brain, increasing ATP consumption; (iii) mitochondrial function and calcium homeostasis are impaired at different levels, thus reducing ATP synthesis; (iv) calpain activation degrades microtubule-associated protein-2, thus altering the microtubular network; and (v) nitric oxide (NO) formation rises, reducing the activity of glutamine synthetase, thus decreasing the elimination of ammonia in the brain (Monfort et al., 2002; Kosenko et al., 2004). In addition, through an excitatory amino acid transporter, ammonia mediates METH-induced increases in extracellular glutamate in order to cause excitotoxicity (Halpin et al., 2014). Interestingly, NMDARs and AMPARs antagonists prevent ammonia-induced toxicity in experimental models (Monfort et al., 2002; Halpin and Yamamoto, 2012).

#### Hydrogen Peroxide

Hydrogen peroxide (H_2_O_2_) is a common neurotoxic agent that induces neurotoxicity in cellular models. Cell lines, including BV-2 microglia, PC12 cells, and SH-SY5Y cells, are ideal for studying H_2_O_2_-induced neurotoxicity. In experimental models, H_2_O_2_-induced neurotoxicity causes mitochondrial dysfunction, oxidative stress, cytotoxicity, apoptosis, and neuronal cell death (Han et al., 2014; Morelli et al., 2014; Ismail et al., 2015; Mesbah-Ardakani et al., 2016; Zhong et al., 2016; Masilamani et al., 2017; Oh et al., 2017) while it also modulates glutamate receptors. Exposure to H_2_O_2_ can activate normally silent NMDARs, potentially via the inhibition of redox-sensitive glutamate uptake. H_2_O_2_ affects synaptic transmission and oxidative stress through the activation of NMDARs (Avshalumov and Rice, 2002). In striatal medium spiny neurons, the generation of AMPAR-dependent H_2_O_2_ is one source of retrograde signals that can block the release of DA (Avshalumov et al., 2008).

#### Cisplatin

Cisplatin (cis-diaminodichloroplatinum) was developed in the 1970s as the first platinum-based antineoplastic agent. Neurotoxicity, a common side effect of cisplatin leads to chemotherapy-induced peripheral neuropathy. An accumulation of cis-platinum in dorsal root ganglion neurons in the form of platinum-DNA adducts is thought to be a key mechanism driving such neurotoxicity (Zhu et al., 2016). In addition, as a known neurotoxic agent, cisplatin is suitable for use in NDD models, because it is known to cause memory and learning impairment through oxidative stress and neuroinflammation (Moneim, 2014; Oz et al., 2015; Zhou et al., 2016; Chen C. et al., 2017). Cisplatin modulates glutamate receptors and induces neural activation through the central upregulation of AMPARs and NMDARs (Holland et al., 2014). NMDARs antagonist protects against cisplatin-induced depression (Lehmann and Kärrberg, 1996).

#### Lead

Lead (Pb) is a well-known neurotoxic agent that brings about cognitive deficits in animal models. As a neurotoxic agent in different study models, Pb caused oxidative stress, synaptic and cholinergic dysfunction, neuroinflammation, autophagy, apoptosis, cognitive deficits, and neuronal cell death (Phyu and Tangpong, 2014; Ye et al., 2015; Chibowska et al., 2016; Meng et al., 2016; Tang et al., 2017; Xue et al., 2017). Pb-induced neurotoxicity may be based on the modulation of glutamate receptors, especially iGluRs. During synaptogenesis, it inhibits the functionality of NMDARs. Moreover, Pb exposure causes a decrease in the expression of AMPAR subunits (GluA1, GluA2, GluA3, and GluA4) with the observed decrease in GluA2 expression being particularly remarkable. Pb-induced neuronal cell death was rescued by three glutamate receptor antagonists: 6-cyano-7-nitroquinoxaline-2,3-dione (CNQX), MK-801, and 1-naphthyl acetyl spermine (a specific Ca^2+^-permeable AMPARs blocker) (Mußhoff et al., 1995; Neal et al., 2011; Ishida et al., 2017).

#### Manganese

Manganese (Mn) can be employed in order to induce neurotoxicity in studies on NDDs, especially PD. Mn-induced neurotoxicity brings about mitochondrial dysfunction, oxidative and nitrosative stress, neuroinflammation, and apoptosis, leading to cell death in different animal and cellular models (Chtourou et al., 2011; Moreno et al., 2011; da Silva Santos et al., 2014; Chen et al., 2016; Li S.J. et al., 2017; Shibata et al., 2017). It alters the functionality of glutamate receptors and it has been reported that Mn inhibits the functionality of NMDAR channels with an implication for psychiatric and cognitive impairment (Guilarte and Chen, 2007). Furthermore, by increasing extracellular glutamate levels and modifying the expression of NMDAR subunits, Mn induces nerve cell damage in mRNAs and proteins in rat striatum (Xu B. et al., 2010). In addition to NMDARs, it may also modulate AMPARs. Co-administration of the excitatory agonist, AMPA, with Mn enhances Mn-enhanced magnetic resonance imaging signals. However, this was attenuated by the co-administration of either the Na^+^ channel blocker tetrodotoxin (TTX), or the broad-spectrum Ca^2+^ channel blocker Ni^2+^ (Wang L. et al., 2015).

#### Mercury

Mercury (Hg) is a naturally occurring heavy metal (Moneim, 2015), and a well-known neurotoxic agent for inducing neurotoxicity. A Hg-induced neurotoxicity model is a powerful tool in the study of neurodegeneration. Hg-induced neurotoxicity in animals causes mitochondrial dysfunction, oxidative stress, neuroinflammation, and apoptosis, overall resulting in cognitive deficits and neuronal cell death (Kempuraj et al., 2010; Ayyathan et al., 2015; Adedara et al., 2016). Hg-induced neuronal death is dependent on glutamate-mediated excitotoxicity. Hg has been found to affect NMDARs by increasing their expression and enhancing their responsiveness. The overactivation of NMDA-type glutamate receptors increases Ca^2+^ influx into neurons, which leads to the activation of important pathways involved in neuronal cell death. Furthermore, Ca^2+^ stimulates ROS generation through the mitochondrial pathway as a result of Hg-induced overactivation of NMDARs that take part in neuronal cell death (Farina et al., 2011; Xu et al., 2012; Moneim, 2015; Engin et al., 2017).

#### Melamine

Another powerful tool in the study of NDDs is melamine-induced neurotoxicity. In studies involving animal models, it has been shown to cause synaptic dysfunction and oxidative stress, leading to cognitive impairment (An et al., 2011, 2012; An and Zhang, 2014). In recent studies, it presynaptically altered the glutamatergic transmission of the hippocampal CA3-CA1 synapses *in vitro*. This alteration is likely linked to a modification to autophagy. Acute melamine exposure impaired spatial memory consolidation by disrupting hippocampal NMDAR-dependent LTD (Zhang H. et al., 2016; An and Sun, 2017).

#### Sodium Azide

NaN_3_ is a neurotoxic agent commonly used for neurotoxicity in both *in vivo* and *in vitro* models. It causes mitochondrial dysfunction, oxidative stress, neuroinflammation, apoptosis, and autophagy, ultimately leading to neuronal cell death (Selvatici et al., 2009; Zhang et al., 2011; Olajide et al., 2016; Shan et al., 2017). Upon the glutamate receptors modulation by NaN_3_ produces neurotoxicity. Studies have found that MK-801 blocks NaN_3_-induced cell death, signifying that NMDARs contributes to mediated cell death (Grammatopoulos et al., 2002; Selvatici et al., 2009).

#### Isoflurane

In experimental animal models, isoflurane brings about mitochondrial dysfunction, neuroinflammation, and apoptosis, leading to cognitive impairments and neuronal cell death (Li et al., 2014; Hu X. et al., 2017; Liang et al., 2017; Su et al., 2017; Wu et al., 2017; Xu et al., 2017). Furthermore, it has been observed to modulate glutamate receptors. One study has reported that NMDAR-mediated excitatory synaptic transmission is more sensitive to isoflurane than non-NMDAR-mediated excitatory synaptic transmission (Xu et al., 2017). In addition, at minimum alveolar concentrations, isoflurane causes GABA_A_ receptor antagonism and increases NMDARs inhibition (Nishikawa and MacIver, 2000).

#### 3-Acetylpyridine

As a potent neurotoxic agent, 3-acetylpyridine (3-AP) readily induces neurodegeneration. 3-AP-induced neurotoxicity causes degeneration in the nigrostriatal dopaminergic system within experimental animal models. Furthermore, 3-AP-induced neurotoxicity can be used for both PD and spinocerebellar ataxia models (Deutch et al., 1989; Weller et al., 1992; Janahmadi et al., 2009; Abbasi et al., 2013; Medina et al., 2015). 3-AP modulates NMDARs and also yields neurotoxicity. Pre-exposure to subtoxic concentrations of NMDA enhances 3-AP toxicity, but the NMDARs antagonists, MK-801 or APV, as well as deprenyl, mazindol, and tetrahydrofolic acid, have no effect on 3-AP toxicity (Weller et al., 1992). Another study has suggested that thyrotropin-releasing hormone receptor agonists ameliorate 3-AP-induced ataxia in rats via NMDARs (Kinoshita et al., 1998).

#### 6-Hydroxydopamine

6-Hydroxydopamine (6-OHDA) is employed to induce PD and study the mechanism of dopaminergic neuron cell death in animal models. It is a selective agent for the nigrostriatal pathway. As 6-OHDA shares certain structural similarities with DA, it can enter dopaminergic neurons via DA transporters, and consequently cause toxicity (Shi et al., 2011). It produces diverse toxic effects, such as mitochondrial dysfunction and oxidative stress, apoptosis, neuroinflammation, and dopaminergic cell death (Chan et al., 2009; Liang et al., 2011; Shi et al., 2011; Thornton and Vink, 2012; Guo et al., 2013; Pyo et al., 2013; Wang J. Y. et al., 2013; Elyasi et al., 2014; Magalingam et al., 2014; Mu et al., 2014; De Jesús-Cortés et al., 2015; Liu et al., 2015; Yan et al., 2015; Zhang et al., 2015; Mirzaie et al., 2016). Disturbance of the dopaminergic system may also cause glutamatergic NMDARs imbalances in the brain (Hallett et al., 2006). In addition, 6-OHDA dysregulates NMDAR functions. NMDARs antagonists increase dopaminergic neuronal survival and prevent a levodopa-induced abnormal motor response (Bibbiani et al., 2005; Yan et al., 2014).

#### Ketamine

Ketamine is considered to be a general anesthetic and is used extensively in pediatric surgery. It is an NMDARs antagonist and is increasingly employed in pre-clinical studies in order to induce psychosis in experimental models (Malhotra et al., 1997; Powers et al., 2015; Wang Q. et al., 2017). As a neurotoxic agent, it is utilized in animal models to induce cognitive impairment. This produces diverse toxic effects such as oxidative stress and apoptosis, leading to cognitive impairment. Ketamine-induced cognitive impairment models have long been employed to study NDDs (Malhotra et al., 1997; Duan et al., 2014; Gazal et al., 2014; Wang et al., 2014; Palsa et al., 2016; Wang Q. et al., 2017). Ketamine-induced neurotoxicity causes a use-dependent blockade of NMDARs. This excitatory synaptic blockade activity possibly causes the loss of responsiveness linked to ketamine anesthesia (Sleigh et al., 2014). Furthermore, in a rat model of ketamine-induced neurotoxicity, extended ketamine exposure produces an increase in the expression of NMDAR (GluN1) (compensatory upregulation). This permits a higher toxic influx of Ca^2+^ into neurons once ketamine is removed from the system, increasing the generation of ROS and neuronal cell death (Liu et al., 2012). In addition, ketamine modulates the AMPARs. However, ketamine-induced inhibition of glycogen synthase kinase-3 contributes to the increase in AMPAR signaling that leads to ketamine antidepressant activity (Beurel et al., 2016). Recently, ketamine-induced impairment of the AMPARs potentiator, PF-04958242, has been shown to affect the verbal learning and memories of healthy volunteers (Ranganathan et al., 2017). In addition, an updated study was carried out with primary cultured cortical neurons and PC12 cells using a ketamine-induced neurotoxicity model (Liu et al., 2011).

#### Colchicine

Colchicine is a plant alkaloid that is regarded as a potent inhibitor of physiological processes. It specifically binds to a receptor site on tubulin and blocks mitosis (Karami et al., 2013). It is one of the major neurotoxins used in the study of AD (Mohamed et al., 2015; Sil et al., 2016b). Colchicine-induced neurotoxicity might be caused by glutamate receptor modulation. Colchicine-induced neuroinflammation leading to neurodegeneration in rats might be linked to NMDARs and therapy with memantine mitigates that toxicity (Sil et al., 2016a).

#### Bisphenol-A

As a neurotoxic agent, BPA mainly affects hippocampal neurogenesis and causes cognitive impairment in animal models (Castro et al., 2013; Tiwari et al., 2016; El Tabaa et al., 2017). Various studies have reported that BPA affects glutamate receptors (NMDARs and AMPARs) and produces neurotoxicity (Xu X. et al., 2010; Xu X.H. et al., 2010; Xu X. et al., 2013). According to a recent study, GluN2A and GluA1 (LTP-related glutamate receptors) were significantly downregulated in BPA-exposed rats (Hu F. et al., 2017). iGluRs that modulate the actions of miscellaneous neurotoxic agents are summarized in **Table 2**.

**Table 2:** iGluRs that modulate the actions of miscellaneous neurotoxic agents.

Toxic agents	Major modulating effects	Reference
Ethanol	Inhibits NMDARs and AMPARs; impairs motor and memory performance	Hicklin et al., 2011; Möykkynen and Korpi, 2012
NH_3_	Activates NMDARs; reduces glutamine synthetase activity; decreases the elimination of NH_3_ in the brain.	Monfort et al., 2002; Kosenko et al., 2004
H_2_O_2_	Activates NMDARs and affects synaptic transmission and oxidative stress	Avshalumov and Rice, 2002
Cisplatin	Upregulates AMPARs and NMDARs; causes neuronal damage	Holland et al., 2014
Pb	Dysregulates AMPARs; causes synaptic dysfunctions and cell death	Mußhoff et al., 1995; Neal et al., 2011; Ishida et al., 2017
Mn	Inhibits NMDARs and AMPARs; causes psychiatric and cognitive impairment	Guilarte and Chen, 2007; Wang L. et al., 2015
Hg	Over activates NMDARs; increases Ca^2+^ influx into neurons	Moneim, 2015
Melamine	Disrupts hippocampal NMDAR-dependent LTD	Zhang H. et al., 2016; An and Sun, 2017
NaN3	Activates NMDARs; causes cell death	Grammatopoulos et al., 2002; Selvatici et al., 2009
3-AP	Activates NMDARs; causes ataxia	Kinoshita et al., 1998
6-OHDA	Activates and dysregulates NMDAR function; causes motor complications and neuronal damage	Bibbiani et al., 2005; Yan et al., 2014
BPA	Alters NMDARs and AMPARs	Xu X. et al., 2010, 2013; Xu X.H. et al., 2010

*6-OHDA: 6-Hydroxydopamine; AMPARs: α-amino-3-hydroxy-5-methyl-4-isoxazolepropionic acid receptors; LTD: Long-term depression; Mn: Manganese; NMDARs: N-methyl-D-aspartate receptors; NH_3_: Ammonia; NaN_3_: Sodium Azide; Hg: Mercury; and H_2_O_2_: Hydrogen Peroxide.*

## Concluding Remarks

Apart from the transgenic model of NDDs, animal and cellular models that make use of neurotoxic agent-induced neurotoxicity are very popular in the study of disease progression and potential therapeutic drugs. However, numerous pharmaceutical research projects have so far failed to discover therapeutic drugs for NDDs using these toxic agent-induced models.

Currently, the use of receptors for targeting drug discovery is an efficient approach. Diversity in the class and structural features of glutamate receptors plays a large role in the pathogenesis of NDDs and serves as a target for drugs for treating neurological disorders. We discussed here numerous neurotoxic agents that are capable of producing neuronal toxicity by altering the functionality of glutamate receptors. Several covered neurotoxic agents produced neuronal toxicity mainly by activating receptors where both ionotropic and metabotropic receptors are important for inducing neurotoxicity. As iGluRs are non-selective cation channels, upon binding to an agonist, they permit the passage of Na^+^ and K^+^, and in certain cases, limited quantities of Ca^2+^. Hence, excitotoxicity that induces neuronal damage might be one of the principal mechanisms of neurotoxic agents. On the other hand, upon an agonist binding to mGluRs, a post-synaptic membrane-bound G-protein is activated. This triggers a second messenger system that opens an ion channel for the mediation of signals. G-protein activation also triggers functional changes within the cytoplasm, culminating in gene expression and protein synthesis and specifically, the activation of diverse signaling systems. The mechanic progression of neuronal damage exerted by neurotoxic agents might be complex because the agents act on metabotropic receptors.

Many studies that use neurotoxic agent-induced models reveal the receptors modulating effects, but do not correlate with associated molecular signaling pathways. Despite knowing the mechanism involving glutamate receptors and neurotoxic agents, future studies focusing on glutamate receptors should still be designed. First, considering the pathological actions of currently available neurotoxic agents, the development of new natural, semi-synthetic, and synthetic neurotoxic agents is recommended. Second, more studies using the potent neurotoxic agents discussed here should be conducted. Third, molecular docking should be performed in order to identify receptor-neurotoxic agent interaction at the molecular level based on updated structural features. Finally, laboratory-based *in vitro* studies in which neuronal cell lines are used should be designed. In addition, *in vivo* studies on model organisms to identify molecular signals that impair by neurotoxic agents should be designed and conducted. By understanding the neurotoxic agent-induced neurotoxicity model, screening of agonist, antagonist and allosteric modulators might be of value for NDD therapy. In summary, by targeting glutamate receptors and their associated signals, a neurotoxic agent-induced neurotoxicity model has the potential to correlate a neurotoxic agent with disease pathophysiology. By considering that pathophysiology, the design and discovery of new and modified therapeutic molecules may be a feasible treatment strategy for NDD therapy.

## Author Contributions

MJ and D-KC conceived and designed the study. MJ performed the literature review, wrote the manuscript, produced the figures, and compiled the table. S-YP performed the literature review and MH edited the data. GK contributed by drawing the figures. I-SK and PG arranged the data. D-KC also supervised and handled correspondence. All authors read and approved the final manuscript.

## Conflict of Interest Statement

The authors declare that the research was conducted in the absence of any commercial or financial relationships that could be construed as a potential conflict of interest.

## Abbreviations

α-Syn: alpha-synuclein
6-OHDA: 6-Hydroxydopamine
AD: Alzheimer’s disease
AMPARs: α-amino-3-hydroxyl-5-methyl-4-isoxazole-propionate receptors
Aβ: amyloid beta
Al: aluminum
BMAA: β-*N*-methylamino-L-alanine
BPA: bisphenol A
DomA: domoic acid
DZP: diazepam
DOX: doxorubicin
DSP-4: [N-(2-chloroethyl)-N-ethyl-2-bromobenzylamine]
GC: glucocorticoid
Hg: mercury
Hcy: homocysteine
GiluRs: ionotropic glutamate receptors
KARs: kainate receptors
LTP: long term potentiation
mGluRs: metabotropic glutamate receptors
MPTP: 1-methyl-4-phenyl-1,2,3,6-tetrahydropyridine
MTEP: 3-[(2-methyl-1,3-thiazol-4-yl) ethynyl]pyridine
Mn: manganese
METH: methamphetamine
NDDs: neurodegenerative diseases
NMDARs: N-methyl-D-aspartate receptors
NaN_3_: sodium azide
PD: Parkinson’s disease
PTZ: pentylenetetrazol
